# The Halitosis Consequences Inventory: psychometric properties and relationship with social anxiety disorder

**DOI:** 10.1038/bdjopen.2018.2

**Published:** 2018-04-06

**Authors:** Mauricio Duarte da Conceicao, Fernanda Salgueiredo Giudice, Lucas de Francisco Carvalho

**Affiliations:** 1 Universidade São Francisco, Rua Alexandre Rodrigues Barbosa, State of Sao Paulo, Brazil; 2 Medical School, Universidade Cidade de São Paulo, Rua Butantã, State of Sao Paulo, Brazil

## Abstract

**Objectives:**

Individuals who complain of halitosis experience psychological consequences that can lead to social, professional, and affective limitations. Research has identified social anxiety disorder (SAD) as the most common psychopathology associated to halitosis complaints. Combining these two lines of research, we sought to determine the validity of the Halitosis Consequences Inventory (ICH), a scale designed to assess the psychological consequences of halitosis complaints. We also investigated the relationship between these consequences and SAD.

**Materials and methods:**

Participants were 436 individuals, including those with and without halitosis complaints (*n*=411 and *n*=25, respectively). Measures administered were the ICH, Social Phobia Inventory and its shortened version, the Liebowitz Social Anxiety Scale, Social Avoidance and Distress Scale, and Fear of Negative Evaluation scale.

**Results:**

The ICH had adequate internal consistency (*α*=0.93) and could accurately discriminate between participants with and without halitosis complaints. Furthermore, individuals with high scores on the ICH were more likely to have SAD.

**Conclusions:**

The ICH is an important tool for determining the aversive halitosis consequences, allowing to identify, with some degree of accuracy, individuals who might require screening for SAD. Besides, there´s a linear relationship between the presence of halitosis consequences and SAD.

## Introduction

Halitosis treatment protocols typically focus on diagnosing and treating symptoms of the condition but not the daily life behavioural, emotional, and cognitive changes of those who suffer from the problem. Aiming to fill this gap, we developed the Halitosis Consequences Inventory (ICH—Inventário de Consequências da Halitose).^1,2^ However, several individuals claiming to have halitosis do not actually meet diagnostic criteria, or their breath malodour is much less severe than they suppose.^3,4^ Studies that have investigated the psychopathological profiles of individuals with halitosis complaints have indicated that social anxiety disorder (SAD) is the one most often associated with this complaint.^5,6^ Thus, the present paper focused on determining the psychometric properties of the ICH (i.e., validity and reliability), as well as investigate its relationship with SAD.

The most commonly used method for halitosis classification, Yaegaki and Coil’s (2000), separates the condition into categories of ‘genuine halitosis’, ‘pseudohalitosis’, and ‘halitophobia’.^4^ However, according to Conceição^7^ and Aydin and Harvey-Woodworth^3^, pseudohalitosis and halitophobia classifications are inadequate.

An important aspect about individuals with pseudohalitosis is that many of them actually have bad breath; however, their halitosis is not perceived on a daily basis because they have an efficient tongue hygiene routine and also a defensive posture when around others.^7^ If these patients were asked to stop cleaning their tongues for 24 h prior to an initial breath assessment, many who were previously classified with pseudohalitosis would actually have genuine halitosis.^7^ Fortunately, this request was recently included in an international consensual protocol for halitosis treatment geared toward general practitioners^8^, thus helping avoid diagnostic errors.

Concerning halitophobia, after treatment, whether for genuine or pseudohalitosis, if patients continue to believe they have halitosis, a reclassification into halitophobia results from failed halitosis treatment. Likewise, halitophobia would suggest an irrational fear but refers to patients believing that treatment was unsuccessful.^3^

The ICH is better suited for classifying halitosis as objective (when halitosis is present and detected) or subjective (when the patient merely complains of having it, but halitosis cannot be detected by medical personnel).^3,7,9–11^ Therefore, the focus groups in the present study are subjects with a halitosis complaint, regardless of whether it is objective or subjective, including individuals who have halitosis but are not aware of that due to olfactory fatigue, and therefore, do not have a halitosis complaint.

Often, patients with halitosis complaint have an unshakable conviction that they have a problem, which can be easily noticed by others.^4,12–14^ In some cases, patients continue to believe that their halitosis persists, despite evidence to the contrary.^1,2,4,14^ Such a belief can have numerous consequences, including feelings of self-depreciation and low self-esteem; social, professional, and affective withdrawal; constant intrusive thoughts of having strong halitosis; interpreting others’ normal gestures and attitudes as if they were expressions of disgust related to their bad breath; and behavioural changes (e.g., talking less or avoiding talking with people who are physically close).^1,2,12–14^ Conceição and Chelegon (2011) termed these consequences ‘behavioural alterations due to halitosis’. We have modified this nomenclature to ‘consequences of halitosis’ in the present study given that these consequences are as much emotional and cognitive as they are behavioural (e.g., misinterpretations of others’ gestures and attitudes).

The ICH was originally developed to assess halitosis psychological consequences. The questionnaire development was based on an analysis of 2500 clinical records from patients at a halitosis clinic between 1998 and 2008. The 18 most frequent changes in behaviours, thoughts, and feelings reported spontaneously by patients attending the clinic were selected.^2^ The 18 changes informed by the patients are:

1. To speak less.
2. To turn the face when talking to someone.
3. To avoid talking near someone.
4. To use breath masking agents.
5. To have thoughts of insecurity related to halitosis.
6. To put the hand over the mouth when talking.
7. To have social restrictions due to halitosis.
8. To have professional restrictions due to halitosis.
9. To have affective restrictions due to halitosis.
10. To hold the breath when talking.
11. To speak less in closed places, such as an elevator or inside a car full of people.
12. To do oral and/or tongue hygiene many times a day.
13. To became socially isolated, avoiding go to appointments or obligations, due to halitosis.
14. To feel devaluated (i.e., feeling of low self-esteem).
15. Behavior changes due to misinterpretation of people’s normal gestures and attitudes, relating them to their offensive breath:
16. Misinterpretation of someone’s act covering his or her nose, correlating this attitude with a change in their breath.
17. Misinterpretation of someone’s act when he/she offers a candy or bubble gum, correlating this attitude with a change in their breath.
18. Misinterpretation that someone they were talking to, steps back a little or turns away while they were speaking, or even gets up when sitting next to them, correlating this attitude with a change in their breath.
19. Misinterpretation that people make comments about their breath.

Each of these 18 changes was operationalised as an ICH item (e.g., ‘Do you talk less because of bad breath?’). All items were dichotomously rated according to the presence or absence of each corresponding consequence. In order to elaborate this research, the ICH questions went through a semantic review, made by a dentist specialised in halitosis treatment and by a psychologist PhD in psychological evaluation, so that the questions could be clearly understood and precisely related to each of the 18 changes in behaviours, thoughts, and feelings that the patients reported.

The only research made with the ICH^1,2^ revealed that among 381 patients with halitosis complaint, 92.38% of them had 7 or more points in the ICH and 63.78%, a score of 13 or more points. On the other hand, 62.99% of the patients had a normal breath or only a slight malodour. That is, the majority of the patients had a conviction of having a severe halitosis, but most of them have not. This result suggests validity evidence based on external criteria, but also indicates the need for psychometric studies accumulating evidences for ICH regarding its clinical adequacy.

The main consequences of subjective halitosis are anxiety symptoms, which are characteristic of certain psychiatric disorders (i.e., SAD/social phobia). As noted above, SAD is the most common mental health disorder associated with halitosis complaint.^5,6,14^ Indeed, Yaegaki *et al.* (1996) reported that strong anxiety exhibited by patients with halitosis complaints could be caused by their social phobia, which suggests that treatment or consultations regarding SAD should be mandatory for both halitophobic and genuine halitosis patients. Zaitsu *et al* (2011) revealed that patients with genuine halitosis who exhibited SAD symptoms tend to have difficulty overcoming their halitosis anxiety, which suggests that treatment for genuine halitosis requires consideration of both breath malodour and SAD. Kursun *et al.* (2014) conducted a study in order to compare the relationship between social anxiety with the pseudohalitosis and genuine halitosis. The Liebowitz Social Anxiety Scale (LSAS) and a questionnaire on halitosis were applied to 100 participants, demonstrating that 62% of them had social anxiety disorder.

In order to verify the correlation between halitosis consequences and SAD, we previously administered the Social Avoidance and Distress Scale (SADS) and Fear of Negative Evaluation (FNE) scale to 164 patients at a halitosis clinic.^1,2^ Results revealed that more than 60% of the sample had a score at or above 14 on the ICH, of which >65% had scores that were ‘high’ on both the SADS and FNE scale.^15^ Thus, we used this sample in the present study.

In this study, the verification of the psychometric properties goes in 2 directions: (1) to determine the sensitivity and specificity of the ICH; (2) to determine the internal consistency of the ICH. Given the clear daily life impact of halitosis and high prevalence of SAD among individuals with halitosis complaints, the present study verified the psychometric properties of the ICH and its relationship with SAD symptoms, making it possible to use this tool in the clinic dental practise, to evaluate halitosis psychological consequences and the possible presence of SAD.

## Methods

### Participants

Subjects for the study were selected from a sample comprised 436 individuals, including 411 with a halitosis complaint (63.7% women; aged 18–74 years; *M*_age_=36.64) and 25 without a complaint (84% women; aged 18–55 years; *M*_age_=26.72). We included a much smaller group without a halitosis complaint in order to increase response variability to the study instruments (around 5% of the total sample).

Among the 411 individuals complaining of halitosis, 164 were selected from a halitosis clinic (compiled from a previous study’s database). The other 247 individuals sample was defined for convenience, selected via the Internet, in response to an advertisement placed on websites related to halitosis area, all of them from the state of Sao Paulo, Brazil. In this group, concerning marital status, 38.5% were single, 46.6% married, 11.3% divorced and 3.6% widower; concerning ethnic origins, 2.43% were asians, 59.11% white, 0.4% indigenous, 9.72% black and 28.34% brown; and concerning the degree of instruction, 3.64% had basic education, 34.01% average education, 44.94% higher education and 17.41% were post graduated. The 25 volunteers without a halitosis complaint were students from a university in the countryside of São Paulo, Brazil. This group was selected randomly from a sample of individuals who did not report a halitosis complaint and had a low ICH score (six or fewer points). After applying these exclusion criteria this group was greatly reduced, from 59 to 25 volunteers. The selection of the volunteers occurred from January to September of 2014.

### Instruments

We included seven instruments for this study: a sociodemographic questionnaire, the SADS, the FNE scale^15^, the self-report version of the Liebowitz Social Anxiety Scale (LSAS-SR)^16^, the Social Phobia Inventory (SPIN)^17^ and its shortened version (Mini-SPIN)^18^, and the ICH.^1,2^ The ICH items are presented in Table 1.

The FNE scale measures discomfort and distress during interpersonal interactions. Specifically, this instrument is used to measure apprehension among individuals when being negatively evaluated, and it includes 30 items with a true–false response format. The SADS scale was developed to quantify social anxiety, and it includes 28 true/false items. The SADS assesses an individual's desires or attitudes regarding escape, avoiding talking with others for any reason, and feeling disturbed, distressed, tense, or anxious during social interactions. The internal consistency for the FNE and SADS is 0.94 analysed by KR-20 (Kuder–Richardson 20 coefficient), and test–retest reliability is 0.78 to 0.94 for the FNE and 0.68 to 0.94 for the SADS.^15^

The LSAS-SR was used to assess typical SAD symptoms and has been validated with Portuguese-speaking samples.^19^ The LSAS-SR is an instrument comprised of 24 items related to two dimensions: fear and avoidance of social situations experienced during the last week. Eleven items are related to social interaction (S) and 13 to public presentation (P), all scored on a four-point Likert scale (0=never, 3=deeply/generally). The total score is calculated by adding the scores obtained from each item, with a maximum score of 72 for the fear dimension and 72 for the avoidance dimension (total max=144). A cutoff of 60 provides the best balance of sensitivity and specificity for classifying participants into generalised SAD. The LSAS-SR provides excellent internal consistency (Cronbach’s alpha=0.90–0.96) and test–retest reliability (Intraclass Correlation Coefficient=0.81; Pearson’s=0.82).

The SPIN assesses the physiological symptoms of fear or flight related to SAD. The SPIN consists of 17 items scored on a five-point Likert scale (0=none, 4=extremely), with a maximum total of 68. We used the Portuguese version of the SPIN.^20^ A cutoff of 19 provides the best balance of sensitivity and specificity. Internal consistency (Cronbach's alpha) ranged from 0.71 to 0.90.

The Mini-SPIN (short SPIN version) is a three-item scale that shows good efficiency as a screening tool for generalised SAD. A cutoff score of six or more points demonstrates a sensitivity of 88.7%, specificity of 90.0%, positive predictive value of 52.5%, and a negative predictive value of 98.5%.^18^ The internal consistency of the Portuguese version has been reported at 0.81.^21^ The inclusion of Mini-SPIN was to evaluate the efficiency of an easy-to-administer scale in patients with halitosis complaint when compared with other SAD scales.

### Procedure

The study was performed in accordance with precepts and regulations for research stated in the Declaration of Helsinki (version 2002). The study was carried out only after obtaining approval from the Human Subjects Ethics Committee of São Francisco University (protocol number CAAE: 36081314.4.0000.5514–10/31/2014). Data were collected by sending participants an invitation, via e-mail, which contained a link to a website featuring the above instruments (hosted by ‘SurveyMonkey’, an online survey software and questionnaire tool). On the first page, volunteers gave their written consent to participate by indicating their acceptance on an informed consent form.

Among the 411 volunteers with halitosis complaints, the 247 individuals recruited through the Internet and 25 volunteers without halitosis complaints were asked to respond to the ICH, LSAS-SR, SPIN, and Mini-SPIN. The 164 remaining volunteers with halitosis complaints (i.e., patients recruited from the halitosis clinic) responded to the ICH, SADS, and FNE scale selected from a database of a previous study.^1,2^

SPSS Statistics 22.0 was used for all data analyses. Variables were handled as ordinal and continuous. We calculated internal consistency values (Cronbach’s *α*) for all instruments; notably, this is the first time that a Cronbach’s *α* for the ICH has been reported. Checking the internal consistency is a minimum condition to demonstrate that the measurement with the test does not present levels that are higher than those that are acceptable from the psychometric point of view.

Participants were separated according to cutoffs of the SAD scales^16–18^, and Student’s *t*-tests were then used to compare means between these groups. For the ICH, we used a cutoff of 14 or greater, as per previous studies using a portion (*n*=164) of the present sample.^1,2^ Effect sizes were calculated using Cohen’s *d*; clinically meaningful results were indicated by a Cohen’s *d* of 0.20 or greater, because 0.20 is generally considered to be a small effect size.

We also performed a receiver operating characteristic (ROC) curve analysis for investigating the ICH’s ability to distinguish individuals with and without halitosis complaints. This analysis, besides enabling the calculation of the area under the curve (AUC), also yields sensitivity and specificity estimates for various cutoffs. The AUC is a measure of how well a parameter can distinguish between two diagnostic groups (diseased/normal). Sensitivity measures the ability of the test to correctly identify individuals with a particular characteristic, while specificity measures the ability of the test to correctly exclude individuals without this characteristic. Considering these two parameters, one can set an ‘optimal’ cutoff. A perfectly accurate test has an AUC of 1.0, while a random guess typically generates an AUC of around 0.5.^22^

## Results

Cronbach’s *α*s for the LSAR-SR, SPIN, Mini-SPIN, and ICH were 0.96 (Fear scale=0.93 and Avoidance scale=0.93), 0.93, 0.78, and 0.93, respectively. As observed in Table 2, the most significant results were observed for the SADS, followed by the LSAS-SR. The same scales demonstrated the greatest magnitude of differences. Similarly, individuals who exceeded the SAD cutoffs consistently reported higher means on the ICH than did those who did not reach these cutoffs. The SADS, followed by the LSAS-SR, showed the largest mean differences between cutoffs. This provides clinically meaningful evidence for patients with higher ICH scores presenting with a higher likelihood of having social anxiety, contributing in the assessment of SAD, but not substituting specific scales in the area, because ICH’s goal is to measure the aversive consequences of halitosis.

**Figure 1:**
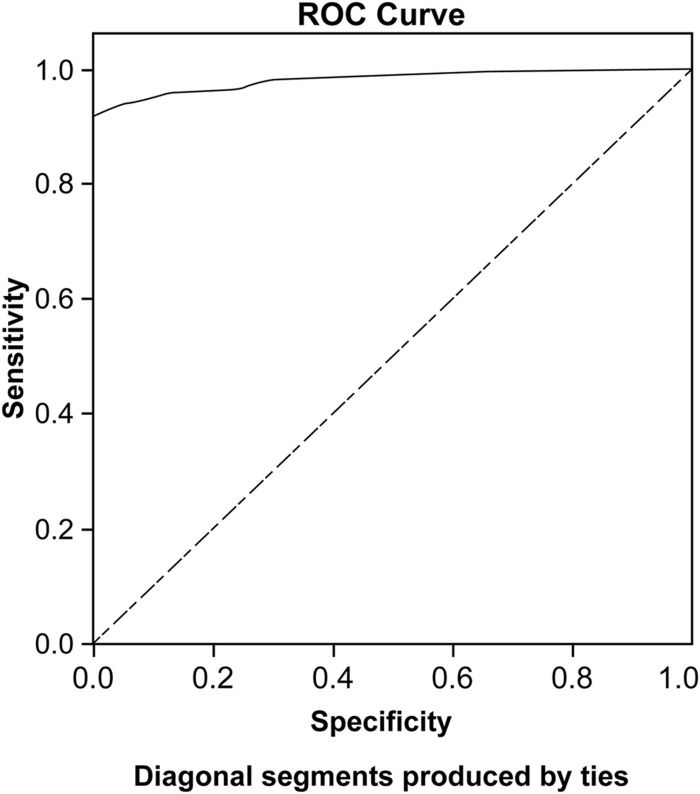
Diagonal segments produced by ties.

We then conducted the ROC curve analysis for predicting how well the ICH distinguishes individuals with (*n*=247) and without halitosis complaints (*n*=25) (Figure 1). The AUC was 0.983 (CI=0.970 and 0.996), which is considered adequate.^22^ According to a visual inspection of the obtained ROC curve indices, the most suitable cutoff corresponds to a gross score of 6.5. At this cutoff, the sensitivity is equal to 95.95% (CI=92.68% to 98.04%), and the specificity is 88% (CI=68.78% to 97.45%).

## Discussion

In line with our study objectives, the instruments used to determine ICH validity based on external criteria revealed adequate levels of measurement error through the use of Cronbach’s *α*.^23,24^ Additionally, results suggest that the ICH predominantly measures consequences of halitosis, given that the ICH’s internal consistency coefficients indicated sufficiently consistent and error-free items.

The Student’s *t*-tests revealed significant differences between the ICH and SAD scales cut-offs. Overall, individuals with high ICH scores were more likely to have higher social anxiety disorder scores.^1,2^ Significance levels and magnitudes were greatest for the SADS, followed by the LSAS-SR, and the lowest for the FNE scale. The highly significant difference for the SADS was probably due to the SADS’ assessment of individuals’ desire to escape and avoid being with or talking to other people, in addition to reporting feelings of being upset, tense, or anxious during social interactions.^15^ These feelings are likely to be present among individuals with a strong belief that others are noticing their halitosis (even if this is not necessarily true). On the other hand, the worse result of the FNE scale probably occurred because it evaluates the concern that individuals feel about the evaluation of others, the distress they suffer for being evaluated negatively and the avoidance of evaluation situations^15^, which shows that people with complaints of having halitosis possibly care more about talking or interacting socially than about being evaluated negatively in general.

To determine the ICH’s validity, we conducted an ROC curve analysis. The AUC of the ICH (0.983) was considered excellent according to Lasko *et al* (2005). In fact, the AUC indicated a near ‘perfect test’, thus demonstrating adequate discriminability between individuals with and without halitosis complaints. Furthermore, the best cutoff score for distinguishing those with and without halitosis complaints was 6.5; this score provided the best balance between sensitivity and specificity, which is useful for diagnostic purposes and results in lower misclassification rates.

In the literature, there are confusions concerning the description and classification about patient’s complaints of having bad breath, when there is no evidence of its presence. That was mainly named pseudohalitosis and also halitophobia. Other names and diagnosis to classify this condition are imaginary halitosis, delusional halitosis, non-genuine halitosis, psychogenic halitosis, psychosomatic halitosis, body odour psychosis, hypochondriasis, olfactory reference syndrome, chronic olfactory paranoid, olfactory obsession, obsessive-compulsive disorder, olfactory delusion and more. Almost all of the terms are psychiatric but they have been described by the dentistry related authors.^10^ The two important prerequisites in the present study to clarify this confusion point and to have a better psychological diagnose are to ask patients to suspend tongue cleaning for at least 24 h before the initial assessment, so as to avoid false negatives during diagnosis, and to regularly administer the ICH and the SAD scales.

Regular clinical administration of the ICH during halitosis treatment could make it possible to measure aversive halitosis consequences. Furthermore, the ICH should be able to identify, with some degree of accuracy, individuals who might require screening for SAD. Thus, besides the diagnosis of objective or subjective halitosis during the initial assessment, the regular use of the ICH and SAD scales by the general dental practitioners could help to diagnose both the presence of the aversive halitosis consequences and social anxiety disorder. This would allow to better comprehend the strong conviction patients have towards their breath malodour, even when halitosis was present and properly treated, in order to determinate whether patients’ treatments would need a psychological and/or psychiatric support or not.

However, the small size of our non-halitosis complaint group is a major limitation of this study. Nevertheless, we believe that this does not diminish the importance the present findings for use in the halitosis field. Future studies should further validate the present findings using a larger sample of individuals without halitosis complaints.

## Conclusion

The Halitosis Consequences Inventory demonstrated adequate internal consistency and an ability to discriminate individuals with and without halitosis complaints. This suggests that the Halitosis Consequences Inventory is an appropriate tool for assessing aversive halitosis consequences. We also observed significant and large differences in social anxiety disorder symptoms between individuals above and below the Halitosis Consequences Inventory cutoffs. This suggests that individuals with high Halitosis Consequences Inventory scores are more likely to exhibit social anxiety disorder symptoms. These findings allow to conclude that there’s a linear relationship between the presence of the consequences of halitosis and social anxiety disorders. However, given the design of the present study, we cannot assert a causal relationship, i.e., whether one of the conditions triggers the other. Thus, it is likely important to screen for social anxiety disorder among these individuals, preferably using easy-to-administer scales.

## Publisher’s note

Springer Nature remains neutral with regard to jurisdictional claims in published maps and institutional affiliations.

## Figures and Tables

**Table 1:** Halitosis Consequences Inventory (ICH)

**Mark YES if you have experienced any of the 18 consequences of halitosis two or more times:**			
		**YES**	**NO**
	1. Do you talk less because of bad breath?		
	2. Do you turn your face when talking to someone because of bad breath?		
	3. Do you avoid talking close to people because of bad breath?		
	4. Do you chew gum, have breath mints, or use mouthwash to mask your bad breath?		
	5. Do you have worries about bad breath (for instance: ‘Do I have bad breath?’ ‘Is it strong?’ and so on)?		
	6. Do you put your hand over your mouth while talking because of bad breath?		
	7. Do you believe that you will be a more spontaneous person in your social life if you stop having bad breath?		
	8. Do you believe that you will be a more spontaneous person in your professional life if you stop having bad breath?		
	9. Do you believe that you will be a more spontaneous person in your affective relationships if you stop having bad breath?		
	10. Because of bad breath, have you ever mumbled (holding your breath) in a situation you had to talk very close to someone?		
	11. Because of bad breath, do you talk less when in closed or crowded spaces such as a car or elevator?		
	12. Because of bad breath, did you start taking better care of your oral hygiene (teeth brushing, flossing, and/or tongue cleaning)?		
	13. Because of bad breath, have you ever given up going out or attending a social event or a commitment?		
	14. If you stop having bad breath, will your self-esteem improve?		
	15. Has someone ever covered his or her nose, and you thought it was because of your bad breath?		
	16. Has someone ever offered you mint drops, and you thought it was because of your bad breath?		
	17. Has someone you were talking to ever stepped back a little or turned away while you were speaking, or got up when you sat next to him or her, and you thought it was because of bad breath?		
	18. Do you believe you have heard comments (indirect and/or by third parties) about your bad breath?		

**Table 2:** Comparison of ICH means based on cut-off score groups for the LSAS-SR, SPIN, Mini-SPIN, FNE, and SADS

	ICH—*M* (s.d.)	*t*	df	*P*	*d*
*LSAS-SR (60)*					
Negative classification of SAD (*n*=160)	12.66 (5.35)	4.268	270	0.001	**0.53**
Positive classification of SAD (*n*=112)	15.20 (3.96)				
					
*SPIN (19)*					
Negative classification of SAD (*n*=120)	12.48 (5.41)	3.693	270	0.001	**0.45**
Positive classification of SAD (*n*=152)	14.67 (4.40)				
					
*Mini-SPIN (6)*					
Negative classification of SAD (*n*=172)	12.84 (5.28)	3.823	270	0.001	**0.48**
Positive classification of SAD (*n*=100)	15.18 (4.03)				
					
*FNE (21)*					
Negative classification of SAD (*n*=93)	13.23 (3.36)	1.830	162	0.069	**0.29**
Positive classification of SAD (*n*=71)	14.23 (3.60)				
					
*SADS (15)*					
Negative classification of SAD (*n*=100)	12.78 (3.60)	4.232	162	0.001	**0.68**
Positive classification of SAD (*n*=64)	15.03 (2.84)				

*Note.* in bold *d*⩾0.20
